# Alcohol Taxes and Birth Outcomes

**DOI:** 10.3390/ijerph7051901

**Published:** 2010-04-27

**Authors:** Ning Zhang

**Affiliations:** Department of Community and Preventive Medicine, The University of Rochester School of Medicine and Dentistry, 601 Elmwood Ave, Box 644, Rochester, NY 14642, USA; E-Mail: ning_zhang@urmc.rochester.edu; Tel.: +1-585-275-0165; Fax: +1-585-461-4532

**Keywords:** alcohol taxes, infant health, low birth weight, APGAR Score

## Abstract

This study examines the relationships between alcohol taxation, drinking during pregnancy, and infant health. Merged data from the US Natality Detailed Files, as well as the Behavioral Risk Factor Surveillance System (1985–2002), data regarding state taxes on beer, wine, and liquor, a state- and year-fixed-effect reduced-form regression were used. Results indicate that a one-cent ($0.01) increase in beer taxes decreased the incidence of low-birth-weight by about 1–2 percentage points. The binge drinking participation tax elasticity is −2.5 for beer and wine taxes and −9 for liquor taxes. These results demonstrate the potential intergenerational impact of increasing alcohol taxes.

## 1. Introduction

Alcohol consumption during pregnancy can lead to adverse health effects for the developing fetus. Because alcohol passes through mothers’ bloodstream into the placenta, it can interfere with fetus’ ability to access oxygen and thereby with nourishment for normal cell development in the brain and other body organs [1]. Prenatal exposure to alcohol may increase the incidence of having an infant with poor health [2–8]. Indicators include: low-birth-weight, which refers to the birth weight lower than 2,500 g. Low APGAR scores are another indicator. The APGAR score is determined by evaluating the newborn baby on five criteria (Appearance, Pulse, Grimace, Activity, Respiration) on a scale from zero to two, then summing up the five values thus obtained. The resulting APGAR scores range from zero to 10; hence, low APGAR scores refer to scores below 7. Researchers have found that no level of alcohol consumption during pregnancy can be deemed entirely “safe,” as even occasional drinking could lead to infant health problems [9–11]. Thus, in 2005, the U.S. Surgeon General reissued the official recommendation (since 1981) that pregnant women not drink alcohol [12].

Various public policies have targeted alcohol consumption among pregnant women. As one example, the expansion of Medicaid eligibility in the late 1980s provided pregnant women with opportunity for more access to medical information regarding the dangers of drinking while pregnant. Subsequent research evaluating that policy yielded conflicting results. Based on data from Tennessee, Pieper *et al.* [13] found no improvement in infant health after the expansion. Gruber and Currier [14], on the other hand, analyzed aggregate state-level data and found the likelihood of LBW decreased in states that adopted the expansion earlier than other states. Another example of public policy in this domain is the Federal Beverage Labeling Act, which mandated warning messages for alcoholic beverage containers addressing the risk of birth defects from alcohol consumption during pregnancy. Research concluded that labeling laws have had little impact on infant health outcomes [15].

Alcohol taxation is another policy approach to discourage drinking and potentially reduce alcohol-related social and health problems, including among pregnant women and their offspring. Although changes in U.S. alcohol taxation have tended to be more the result of efforts to balance state revenues than to improve the public health [16], research demonstrating the perils of prenatal alcohol exposure for developing fetuses has led some public health and policy experts to advocate for more frequent adjustments (upward) in alcohol taxes [17–19]. The potential effect of such taxation on infant health outcomes has received little attention in the policy literatures, however. This study is the first to show that raising alcohol taxes could have a beneficial impact on infant health. What have heretofore been unintended intergenerational effects could provide additional incentive to reform alcohol taxation and policies in the U.S.

## 2. Methods

### 2.1. Estimation Models

In his seminal work, Becker [20] established a theory whose utility function of altruistic parents includes children’s health. Based on this theory, Rosenzweig and Schultz [21] developed a hybrid equation, upon which this study builds to account for the relationship between maternal drinking and infant health:

(1) C=F(D,Z;μ)

where infant health (*C*) is a function of alcohol drinking during pregnancy (*D*), consumption of healthy-good (*Z*), and health endowment (*μ*). Without losing generosity, assume that alcohol consumption during pregnant has a negative impact (*F_D_* < 0), and healthy-good consumption has a positive impact (*F_Z_* > 0), on infant health. Also assume that *F_μ_* > 0. Hence, the demand function for alcohol is:

(2) $$D=f(P_{A},Y,X;\mu )$$

which is determined by alcohol price (*P_A_*), income (*Y*), demographic factors (*X*) and unobserved individual factors. The demand function for health-good is defined similarly, *i.e.*, *Z* = *f*(*P_Z_, Y, X; μ*), where *P_Z_* is the price of healthy-good, and other notations are the same as in equation (2). Thus, substituting demand functions of both alcohol and healthy-good into equation (1), with a normalized price (*P_Z_* = 1), a reduced-form model of child health is:

(3) $$C=F(P_{A},Y,X;\mu )$$

Researchers have found that alcohol taxes (T) can well represent alcohol prices [22]. Hence, Equation (3) changes to:

(3′) C=F(T,Y,X;μ)

Equation (3′) shows the direct impact of increase in alcohol taxes on infant health. Many researchers used beer tax alone as an explanatory variable, for the reason that all U.S. states apply specific excise taxes to beer and taxes on wine and distilled spirits in license states [22]. However, reports have shown that wine and liquor consumption increased significantly over the past few decades [23]. Additional evidence indicates that more and more Americans enjoy wine and distilled liquor, and even prefer wine to beer [24]. Further, while beer is more favored by men, wine and liquor tend to be favored by women [25]. Therefore, this study distinguishes taxes on all three types of alcohol: beer, wine and liquor. Specifically, the estimation is based on Equation (3′):

(4) $$C_{tst}=\beta_{1}X_{t}+\beta_{2}T_{jst}+\lambda_{1s}+\lambda_{1t}+\xi_{1tst},$$

(5) $$D_{tst}=\delta_{1}X_{t}+\delta_{2}T_{jst}+\lambda_{2s}+\lambda_{2t}+\xi_{2tst}$$

where *i*, *s*, and *t* index individual, state and conception year, respectively. Taxes on beer, wine and liquor taxes (*i.e.*, *T*_j_) are included one-by-one into the model to avoid the multicollinearlity problem. *λ*_1_ and *λ*_2_ control for state- and year-fixed-effects. *ξ* is a random error with zero mean and finite variance. When outcome variables are continuous, an ordinary-least-square estimation is estimated; when they are discrete, a probit model is examined. One could argue that even if the effects of the alcohol taxes on women’s behaviors and on birth outcomes are consistent with our hypothesis, other factors, such as macroeconomic conditions, could drive the improvement. To remove this concern, the estimation model includes real per capita income representing state-specific macroeconomic environments during the birth years studied.

### 2.2. Data

Estimation of birth outcomes (Equation (4)) is based on data from the Natality Detailed Files (1985–2002), a U.S. census of births derived directly from birth records. Infant health, mothers’ education, marital status, age, and race/ethnic group were included. Fathers’ information is available only for some years and thus was excluded. Samples were restricted to children whose health outcomes were available. In addition, children whose mothers were older than 44 years were excluded since decision of having a child for these women may be different from mothers with regular ages. The conception year in the Natality files is coded according to the month of the last menstrual period. When last menstrual period was missing, infants’ birth dates and a clinical estimate of gestation length were used.

Since 1989, most states have required that mothers report drinking behaviors during pregnancy in the birth certificate. However, alcohol consumption is generally under-reported. Only around 2% of mothers reported that they drank during pregnancy in birth certificate data, *versus* the estimated 12% of pregnant women nationwide who actually did [26]. Therefore, mothers’ alcohol drinking in the Natality files was not employed in the estimation models. Instead, drinking behaviors (Equation (5)) were estimated based upon the Behavioral Risk Factor Surveillance System (BRFSS; 1985 to 2002), an ongoing nationally representative survey of the non-institutionalized US population age 18 years and older. Data in even years from 1994 to 2000 were dropped from the sample since only 10 states employed the alcohol module for those years. Only pregnant women were included in our sample and the survey year represented the conception year.

Variables of alcohol consumption were constructed as follows: First, alcohol consumption was set to 1 if the respondent drank during the previous 30 days, and 0 if not. This question was then justified using a subsequent question regarding the number of alcoholic drinks consumed in total. Table 1 defines the variables. For babies born from 1985–2002, the average birth weight was 3,326 grams; 7% of babies were low birth-weight (LBW); 1% extremely low birth-weight (< 1,500 g, ELBW); and another 1% had low APGAR scores. On average, 12% of pregnant women drank alcohol. Asked about drinking “during the previous 30 days,” most of these women reported consuming about four drinks; 1% reported binge drinking.

Alcohol taxes were derived from the Brewing Industry of the United States and National Conference of State Legislatures. In this paper, alcohol taxes combined federal and state taxes (adjusted to 1982–1984 CPI). Federal tax rates have been stable in nominal terms since 1951. The only increase in federal taxes on beer and wine occurred in 1991, when beer taxes doubled from 16 to 32 cents per six pack and wine taxes jumped from just over 3 cents to about 21 cents per 750 milliliter bottle. In 1985, tax rates on distilled spirits rose from $12.5 to $13.5 dollars per proof gallon [27]. State taxes increase more frequently such that there are variations across states. Appendix A shows the year when states last raised alcohol taxes. In a state where alcoholic beverages, particularly distilled liquors, are sold through state stores, taxes combine specific, ad valorem and implicit taxes. In the estimation, taxes are missing if it is a monopoly controlled state. Under current laws, the way in which alcohol taxes are levied involve different measures for different types of alcohol [28]. Liquor and distilled spirits are measured per proof gallon, per barrel for beer and per gallon for wine. Hence, taxes are much lower on the alcohol content of beer and wine than on the alcohol content of liquor/distilled spirits. Over this period (1985–2002), the average beer tax was 60 cents per six pack, wine tax $1 per 750 mL bottle and liquor tax $16 per proof gallon. Alcohol taxes were merged with both datasets, respectively, based on conception year.

## 3. Results and Discussion

Table 2 reports the relationship between alcohol taxation and drinking during pregnancy. Models in Columns 1–3, which aim to show the direct impact of alcohol taxation on infant health, do not include maternal information. Models in Columns 4–6 include maternal characteristics, demonstrating whether adding mothers’ characteristics and thus demonstrate whether inclusion of maternal variables affects our understanding of the potential impacts of alcohol taxes on women’s behaviors and on subsequent newborn health. If results across models do not vary, the estimation is robust to model specification, meaning that alcohol taxes do impact infant health, even while controlling for mothers’ characteristics. The estimated marginal impacts of taxes on drinking prevalence were negative but statistically insignificant. The probability of binge drinking dropped by 3% points per one-cent increase in beer taxes, 1.5% points for an equivalent increase in wine taxes, and 0.6% points for liquor taxes, and these results do not change across models. These results are statistically significant, which suggests that there exist negative impacts of alcohol taxes on heavy drinking among pregnant women. The coefficient of liquor taxes is small while reasonable since the unit of taxes variables is “cent” (to be consistent across three types of taxes) while the mean of liquor taxes is $15, or 1,500 cents. The effect of taxes on quantity of drinks, however, decreased as more controls were included, implying that quantity of consumption is not fully determined by tax-effect, as mothers’ demographic characteristics also have an effect. Given the low incidence of binge drinking (1%), I have run linear probability and logit models as well. Results from these two estimations are similar to the Probit model presented here, except that clustered standard errors are a little different.

Price elasticity of demand measures the responsiveness of the quantity demanded of a good or service to a change in its price. Following Evans and Ringel [29], tax elasticity of demand changes on drinking participation is: $ɛ=\frac{\delta D}{\delta T}\times \frac{\tau }{D}$. Specifically for this paper, $ɛ=\delta_{2}\times \frac{\tau }{\bar{D}}$ where *T̄* and *D̄* are average level of taxes and average probability of drinking while pregnant. Table 2 also presents the tax-induced elasticities of alcohol demand. Per any 1% increase in taxes, the drinking prevalence decreases by 0.1–0.2%, indicating that drinking participation is irresponsive to prices induced by taxes among pregnant women. With the same amount of changes in taxes, the quantity of alcohol consumption decreases by 1.2–1.8%, meaning that quantity of demand is sensitive to alcohol taxes. It is not surprising that the quantity elasticities are larger than participation elasticities, as theory predicts that quantity elasticities encompass both the participation and quantity responses. With this similar logic, tax elasticities of binge drinking are also larger than participation elasticities: the binge drinking decreases by 2.5% with 1% increase in beer or wine taxes, and by 9–10% with liquor taxes. These estimates show that binge drinking is very sensitive to prices among pregnant women, and most sensitive to liquor taxes. Moreover, these elasticities among pregnant women are larger than those in the general population, in which elasticities range from −0.92 to −2.24 [27].

There are two ways that alcohol taxes can be seen to impact birth outcomes. One is through their potential effect on the types of women who give birth. Studies have shown a positive association between alcohol use and risky sexual behavior, especially in teenagers [30–31]. Therefore, a tax hike may reduce the likelihood of youth engaging in unprotected sexual activities, which delays motherhood. In this study, such impact is examined by characterizing the mothers shown in Table 3. The proportion of mothers younger than 24 years decreased by 16% points per one-cent increase in beer taxes, 2% points in wine taxes and 0.01 in liquor taxes. The relative number of mothers between ages 24–35 likewise increased with raises in alcohol taxes. Moreover, any one cent increase in beer taxes decreased the probability of a mother with an education level of high school dropout or lower (*i.e.*, years of education smaller or equal to 12) by 29% points, 7% points in wine taxes, and 1% point in liquor taxes. As children born to these mothers are at higher risk for health problems [32], a decreasing proportion of these women implies better birth outcomes with higher alcohol taxes.

Table 4 shows the second type of effect, *i.e.*, alcohol taxes impact average infant health. Mothers’ characteristics are important factors for infant health since results change when models included mothers’ features. On average, a one-cent hike in beer taxes increased the average birth weight with 1grams, 0.2–0.3 grams in wine taxes and 0.07 grams in liquor tax. It is also evident that alcohol taxes lead to a reduction of LBW. The likelihood of LBW decreased with 1∼2% per one-cent raise in beer taxes, 0.2–0.3% points in wine taxes and 0.1% points in liquor taxes. Results are all statistically significant, indicating that alcohol taxes have positive impacts on improvement of birth weight or reduction of LBW. Effects of these policies on ELBW and low APGAR scores were also investigated. The incident of ELBW reduced with 0.1–0.3% points per one-cent increase in beer, wine or liquor taxes. The estimation on low APGAR scores is not inclusive. Increases in beer or wine taxes raised the incidences of low APGAR scores by a small amount. While there is no obvious explanation for this finding, one possible reason is that infants with a low APGAR score may be dead and were not included in birth certificate data. Another possible reason may be the low average prevalence of low APGAR scores (1% overall, as shown in Table 1).

## 4. Conclusions

Women not only are more vulnerable than men to alcohol’s effects, but also can pass on adverse consequences of alcohol use to the developing fetus if they drink while pregnant [33]. This study examines the relationships between increased alcohol taxes, pregnant women’s drinking behaviors, and infant health. Unlike tobacco taxes, which vary based largely on public health concerns, state changes in alcohol taxation have tended to be made for more clearly financial reasons; public health benefits, in the absence of a state budget deficit, are not sufficient incentives for a state to raise alcohol taxes [34].

The key findings from this study show that any one-cent increase in alcohol taxes decreases the prevalence of LBW by 0.1∼2% points. Since our estimates are based on census data, which include 70 million babies, the results indicate that up to 98,000 fewer babies would have been born at LBW over these years had there been a one-cent increase in alcohol taxes, based on the mean of effects of beer taxes. Since LBW is related to other health and socio-educational consequences for children, raising alcohol taxes could confer significant public health and associated benefits. Findings also show that quantity and binge drinking participation are sensitive to alcohol prices (particularly, as related to liquor taxes) among pregnant women, and that these elasticities are larger than those for the general population. To assure robustness of our findings, we utilized taxes for the pre-and-post-conception years, respectively, in our models. Magnitudes of all coefficients became 100 times smaller with no significance so that the identification assumption is correct. Results are available upon request.

The primary limitation of this study is that it remains unclear whether the mechanism by which increased alcohol taxes predict infant health occurs solely through a reduction of binge drinking; unfortunately, no single dataset includes reliable information on both birth outcomes and drinking during pregnancy. Nevertheless, the estimates drawn from the national representative data from BRFSS confirm that lower drinking prevalence, especially heavy drinking, during pregnancy is an important factor that contributes to better birth outcomes. Future work should address whether this relationship is causal, perhaps through development of a dataset with both birth outcomes and drinking together.

## Figures and Tables

**Table 1.** Variables definition and descriptive stats.

**Variable**	**Descriptive States**	**Mean (sd)**
A. Birth Outcomes (Natality Detailed Files, 1985–2002) ^*^		
Birth weight	Birth weight in grams	3,329.7 (605.59)
Low birth weight	Indicator variable (1 if birth weight < 2,500 grams and 0 otherwise)	0.07 (0.30)
Extremely low birth weight	Indicator variable (1 if birth weight < 1,500 grams and 0 otherwise)	0.01 (0.12)
Low APGAR scores	Indicator variable (1 if APGAR score < 7)	0.01 (0.12)

B. Drinking Behaviors (BRFSS, 1985–2002) ^**^		
Drinker	Indicator variable (1 if a pregnant woman drank during the past 30 days)	0.127 (0.4545)
Drinks/month	Average number of drinks during the past 30 days for pregnant women	4 (20.34)
Binge drinker	Indicator variable (1 if a pregnant women drank at least 5 drinks per occasion)	0.014 (0.1192)

C. Alcohol Taxes (1982–1984 US Dollars) ^***^		
Beer tax	combined federal and state tax on beer per gallon in dollar	0.6 (0.28)
Wine tax	combined federal and state tax on wine per gallon in dollar	1.02 (0.53)
Liquor tax^§^	combined federal and state tax on liquor per proof gallon in dollar	15.78 (6.58)

* There are 71,501,237 infants with birth weight and 55,054,916 with APGAR scores.

** Data from 1994, 1996, 1998 and 2002 were dropped because only 10 states employed the alcohol module in BRFSS for those years.

*** In a state where alcoholic beverages, particularly distilled liquors, are sold through state stores, taxes combine specific, ad valorem and implicit taxes. Taxes are missing if it is a monopoly control state.

§ Taxes are much lower on the alcohol content of beer and wine than on the alcohol content of liquor or distilled spirits because the taxes are determined on the basis of different liquid measures. Liquor is measured in proof gallons (a standard unit for measuring the alcohol content of a liquid). Beer is measured by a barrel and wine is measured by a gallon.

**Table 2.** Alcohol Taxes and Drinking while pregnant (BRFSS).

**Variables**	**Drink (0/1) (1)**	**# Drinks last month (2)**	**Binge drinks (0/1) (3)**	**Drink (0/1) (4)**	**# Drinks last month (5)**	**Binge drinks (0/1) (6)**
A. Coefficients						
Beer tax	−0.003 (0.006)	−0.2338 (0.247)	−0.033*** (0.018)	−0.004 (0.006)	−0.164 (0.243)	−0.031** (0.016)
Wine tax	−0.001 (0.002)	−0.1133* (0.092)	−0.015*** (0.007)	−0.002 (0.002)	−0.097 (0.089)	−0.016*** (0.005)
Liquor tax	−0.0003 (0.001)	−0.0151 (0.026)	−0.005*** (0.002)	0.0002 (0.001)	−0.0109 (0.023)	−0.006*** (0.002)

B. Elasticity (Absolute Value)						
Beer tax	0.238	1.244	2.499	0.329	0.875	2.462
Wine tax	0.127	1.284	2.493	0.317	1.098	2.661
Liquor tax	0.147	1.82	8.975	0.419	1.319	10.406
Covariates	no	no	no	yes	yes	yes

Data come from the BRFSS (1985–2002). Even years between 1994 and 2000 were dropped because of its lack of national representative. Models in Columns 4–6 include covariates such as mothers’ age, age square, educational attainments, race/ethnic group, marital status and real income per capita. All taxes and prices are in cents and adjusted to CPI 1982–1984. In all models, state- and year-fixed effects are included. Marginal effects are reported with state-year clustered errors underneath in the parentheses.

*** significance at 1% level,

** significance at 5% level, and

* significant at 10% level. The sample sizes are as following:

**Table:** 

	Sample size

Beer tax	17,242
Wine tax	15,945
Liquor tax	11,411

**Table 3.** Composition of Mothers (Natality Files).

**Variable**	**Young mother (< 24)**	**Mother (25–35)**	**Low education**
**Beer tax**	−0.164^***^ (0.015)	0.145^***^ (0.010)	−0.289^***^ (0.075)
**Wine tax**	−0.017^***^ (0.005)	0.023^***^ (0.003)	−0.067^**^ (0.026)
**Liquor tax**	−0.001 (0.002)	0.008^***^ (0.001)	−0.009^*^ (0.006)
	# obs in estimation including beer taxes:		67,644,465
	# obs in estimation including beer taxes:		64,013,352
	# obs in estimation including beer taxes:		49,787,719

Note:

1. Models include covariates such as mothers’ educational attainments, race/ethnic group, marital status and income per capita. All taxes and prices are in cents and adjusted to CPI 1982–1984.

2. In all models, state- and year-fixed effects are included. Marginal effects are reported with state-year clustered errors underneath in the parentheses.

*** significance at 1% level,

** significance at 5% level, and

* significant at 10% level.

3. The number of observations regarding to models including taxes on beer, wine and liquor, respectively, are as follow:

**Table:** 

	Models on birth weight, LBW and ELBW	Models on APGAR scores

Beer tax	67,644,465	52,086,168
Wine tax	63,964,676	48,420,369
Liquor tax	497,693,317	34,284,469

**Table 4.** Alcohol Taxes and Infants Health (Natality Files).

**Variable**	**Birth weight (grams) (1)**	**LBW (0/1) (2)**	**ELBW (0/1) (3)**	**Low APGAR score (0/1) (4)**	**Birth weight (grams) (5)**	**LBW (0/1) (6)**	**ELBW (0/1) (7)**	**low APGAR score (0/1) (8)**
**Beer tax**	0.636*** (0.002)	−0.013*** (0.001)	−0.003*** (0.0004)	0.0005* (0.0004)	0.931*** (0.003)	−0.023*** (0.001)	−0.002*** (0.0004)	−0.0002*** (0.00001)
**Wine tax**	0.212*** (0.008)	−0.003*** (0.0003)	−0.002*** (0.0002)	0.0005*** (0.0002)	0.340*** (0.006)	−0.006* (0.0004)	−0.002** (0.001)	0.0002 (0.0002)
**Liquor tax**	0.071*** (0.002)	−0.001*** (0.0001)	−0.0001*** (0.0000)	−0.0001** (0.0006)	0.072*** (0.027)	−0.001*** (0.0001)	−0.001 (0.001)	−0.0001*** (0.0000)
**Covariates**	no	no	no	no	yes	yes	yes	yes

1. Models in Columns 4–6 include covariates such as mothers’ age, age square, educational attainments, race/ethnic group, marital status and income per capita. All taxes and prices are in cents and adjusted to CPI 1982–1984.

2. In all models, state- and year-fixed effects are included. Marginal effects are reported with state-year clustered errors underneath in the parentheses.

*** significance at 1% level,

** significance at 5% level, and

* significant at 10% level.

3. The number of observations regarding to models including taxes on beer, wine and liquor, respectively, are as follow:

**Table:** 

	Models on birth weight, LBW and ELBW	Models on APGAR scores

Beer tax	67,644,465	52,086,168
Wine tax	63,964,676	48,420,369
Liquor tax	497,693,317	34,284,469

## Appendix A. State alcohol taxes last raised

**Table:**

State	Alcohol taxes last raised	State	Alcohol taxes last raised
Alabama	1982	Nevada	2003
Alaska	2002	New Hampshire	1991
Arizona	1983	New Jersey	1992
Arkansas	2001	New Mexico	1993
California	1991	New York	20019
Colorado	1976	North Carolina	1969
Connecticut	1989	North Dakota	1967
Delaware	1990	Ohio	1993
District of Columbia	1989	Oklahoma	1987
Florida	1999	Oregon	1977
Georgia	1964	Pennsylvania	1947
Hawaii	1998	Rhode Island	1989
Idaho	1961	South Carolina	1969
Illinois	1999	South Dakota	1988
Indiana	1981	Tennessee	2002
Iowa	1986	Texas	1984
Kansas	1987	Utah	2003
Kentucky	1982	Vermont	1981
Louisiana	1948	Virginia	1993
Maine	1986	Washington	1997
Maryland	1972	WestVirginia	1966
Massachusetts	1975	Wisconsin	1969
Michigan	1966	Wyoming	1935
Minnesota	1987		
Mississippi	1986		
Missouri	1971		
Montana	1992		
Nebraska	2003		

Note: The year is recorded when beer, wine or liquor taxes were raised. In most states, the three taxes changed at the same time.
